# Left Ventricular and Atrial Deformation in Patients with Acute Decompensated Heart Failure: A Pilot Study

**DOI:** 10.3390/diagnostics14131368

**Published:** 2024-06-27

**Authors:** Jakub Jurica, Martin Jozef Péč, Marek Cingel, Tomáš Bolek, Marianna Barbierik Vachalcová, Simona Horná, Peter Galajda, Marián Mokáň, Matej Samoš

**Affiliations:** 1 Department of Internal Medicine I, University Hospital Martin, Jessenius Faculty of Medicine in Martin, Comenius University in Bratislava, Kollárova 2, 036 59 Martin, Slovakia; kubo.jurica@gmail.com (J.J.); pec5@uniba.sk (M.J.P.); marek.cingel1@gmail.com (M.C.); ato.bolek@gmail.com (T.B.); horna.simona@gmail.com (S.H.); peter.galajda@uniba.sk (P.G.); mokanmarian@gmail.com (M.M.); 2 Department of Cardiology, Teaching Hospital in Trenčín, 911 01 Trenčín, Slovakia; 3 Department of Cardiology I, Faculty of Medicine, P.J. Šafárik University in Košice and East-Slovakian Institute of Heart and Vessel Diseases (VÚSCH, a.s.) in Košice, 040 11 Košice, Slovakia; marianna.vachalcova@gmail.com; 4 Division of Acute and Interventional Cardiology, Department of Cardiology and Angiology II, Mid-Slovakian Institute of Heart and Vessel Diseases (SÚSCCH, a.s.) in Banská Bystrica, 974 01 Banská Bystrica, Slovakia

**Keywords:** global longitudinal strain, reservoir strain, speckle-tracking echocardiography, acute decompensated heart failure

## Abstract

Aims: The aims of this study were to compare global longitudinal strain of the left ventricle (LV-GLS) and reservoir strain of the left atrium (R-LAS) values between patients with acute decompensation of chronic heart failure (HF) and a control group. Methods: Sixteen patients admitted to our ward for acute decompensation of HF were enrolled in this study. Transthoracic echocardiography (TTE) with two-dimensional speckle-tracking analysis (2D ST) was performed in each patient. The patients were divided into two subgroups according to the value of left ventricular ejection fraction (EF) using a cut-off value of ≤40% to distinguish heart failure with reduced ejection fraction (HFrEF) from heart failure with preserved ejection fraction (HFpEF). The control group consisted of 16 individuals without a history of cardiovascular disease, each of whom underwent 2D ST analysis as well. Results: We found that LV-GLS and R-LAS were significantly lower in both the HFrEF and HFpEF subgroups in comparison with the control group (LV-GLS: −13.4 ± 4.7% vs. −19.7 ± 2.5%, *p* ˂ 0.05; R-LAS: +12.2 ± 6.9% vs. +40.3 ± 7.4%, *p* ˂ 0.05). Furthermore, there was a significant difference in LV-GLS (−9.6 ± 3.2% vs. −15.2 ± 4.3%, *p* ˂ 0.05) but not in R-LAS (+13.7 ± 8.6% vs. +11.4 ± 6.2%) between the HFrEF and HFpEF subgroups. Conclusions: Our study demonstrated a significant difference in LV-GLS and R-LAS in all enrolled HF patients compared to the control group. There was also a significant difference in LV-GLS between the HFrEF and HFpEF subgroups.

## 1. Introduction

Two-dimensional speckle-tracking analysis (2D ST) is an echocardiographic method that provides an assessment of myocardial deformation and can be used as a diagnostic tool to determine the function of the left ventricle (LV) and the left atrium (LA). Two-dimensional ST is based on detecting a certain object in an echocardiographic image and tracking its change of position during the movement of the heart wall over the course of a heart cycle. In this way, it is able to analyze and determine the deformation parameters of the heart wall during this cycle. The percentage change in the position of this object is expressed as strain, which stands for the unit of 2D ST. Two-dimensional ST can be applied to any heart chamber, but in current clinical practice, the global (endocardial) longitudinal strain of LV (LV-GLS) and the reservoir strain of LA (R-LAS) are the two most widely used parameters. LV-GLS, i.e., the shortening of LV in a longitudinal direction during systole, assesses the systolic function of LV [1]. Its clinical importance comes from the fact that it provides a means of detecting subclinical myocardial dysfunction of LV, while left ventricular ejection fraction (LV EF) might still be considered normal at that stage. For instance, this proves particularly useful in cardio-oncology, where LV-GLS has been shown to detect chemotherapy-related cardiac dysfunction at an earlier stage than EF [2].

With respect to R-LAS, it represents one of three phases of the LA cycle, during which LA fills up with blood and expands from the minimum size just after the closure of the mitral valve to its maximum size prior to the next opening of the mitral valve. R-LAS has the greatest clinical importance of the three phases as a decrease in R-LAS below 35% indicates diastolic dysfunction of LV [3]. Its value is also indirectly proportional to the severity of diastolic dysfunction, which explains the focus of recent studies on this parameter yielding results that consider R-LAS a better diagnostic marker for HFpEF than the standard echocardiographic parameters [4]. Moreover, R-LAS is subject to ongoing research about its potential to become a predictor for the risk of thromboembolic complications in atrial fibrillation alongside the CHA_2_DS_2_-VASc score and the potential to predict the effect of control of rhythm strategies, i.e., radiofrequency ablation or electrocardioversion, in the management of atrial fibrillation [5].

As there are limited data regarding the use of 2D-ST assessment in patients with acute decompensated HF, the aim of this study was to assess R-LAS and LV-GLS at the time of acute decompensation of HF.

## 2. Methods

### 2.1. Study Design

We conducted a pilot, prospective and observational study. The study protocol was reviewed and approved by the local ethics committee. This study was performed during the period from 1 January to 30 May 2023. During the study period, consecutive patients admitted to wards at the Department of Internal Medicine I, University of Martin, for acute decompensated heart failure were enrolled in the patient group. Patients were enrolled if they met the following criteria:

History of chronic heart failure fulfilling the ESC diagnostic criteria [6];
Presence of HF signs and symptoms severe enough to seek urgent medical aid (not manageable with short-term ambulatory intravenous diuretic therapy);
NT-proNBP levels ≥ 125 ng/L on admission;
Good imaging quality on echocardiography allowing for the performance of 2D ST analysis;
No end-stage kidney or liver disease, recent (last 6 months) or disabling stroke or active malignancy.

After enrolment, written informed consent was obtained for study participation. All the enrolled patients underwent study echocardiographic examination for 2D ST analysis on day 1 of their in-hospital stay.

During in-hospital stay, all patients received intravenous loop diuretic therapy. Patients with systolic blood pressure ≥ 110 mmHg received intravenous vasodilators; patients who presented with hypotension and/or tissue hypoperfusion on admission received intravenous inotropic therapy (dobutamine). Discretion as to the administration of intravenous digoxin and other heart failure therapies (including chronic therapy) was left to the attending physician. After decongestion, patients were switched to oral diuretics and chronic HF therapy was up-titrated according to ESC guidelines [6] and local pharmacotherapy policy (see Section 5).

For the final analysis, patients were divided into 2 sub-groups according to the value of LV EF using a cut-off value of ≤40% to distinguish heart failure with reduced ejection fraction (HFrEF) from heart failure with preserved ejection fraction (HFpEF).

Consequently, a control group of healthy individuals was created. Individuals were eligible for enrolment in the control group if they met the following criteria:

No history of cardiovascular disease;
No medication affecting the cardiovascular system;
No family history of cardiovascular disease before the age of 65 years;
Normal serum NT-proBNP levels;
Sinus rhythm with no abnormalities on ECG;
No evidence of structural heart disease on echocardiography.

All controls signed written informed consent to study participation and underwent echocardiographic examination for assessment of 2D ST.

### 2.2. Echocardiography and 2D ST Analysis

Echocardiography was performed on day 1 of patients in-hospital stay by two experienced examinators (M.S. and T.B.) who were blinded to the patients’ clinical data and outcomes at the time of examination. Standard images in standard views were acquired and measured according to the recommendations of the American Society of Echocardiography and European Association of Cardiovascular Imaging [7,8]. All transthoracic echocardiography and speckle-tracking strain imaging were performed using one ultrasound machine (Vivid^®^ E95, GE Medical Systems, Milwaukee, WI, USA) with a 1.4–4.6 Hz transducer. LV end-diastolic dimension and wall thickness were measured using M-mode, and M-mode echocardiography was conducted in the parasternal long axis (PLAX) view. LV mass index was calculated using a validated linear method and indexed according to the body surface area (BSA). LA volume index was assessed in apical 4-chamber (A4C) and apical 2-chamber views (A2C) and indexed to the BSA. LV EF was assessed using the automated Simpson’s biplane formula (AutoEF^®^ software, GE Medical Systems, Milwaukee, WI, USA) on A4C and A2C views. As mentioned, LV EF ≤ 40% was used as a criterium for reduced LV EF. LV filling was assessed by pulsed wave Doppler echocardiography from trans-mitral flow and by pulsed tissue Doppler echocardiography from mitral annular (septal and lateral) position. The peak E wave velocity and the average of peak diastolic velocities of the mitral septal and lateral annulus (E’) were measured. E/E’ ratio was calculated as a parameter of LV filling pressure. E/E’ > 9 was defined as an indirect sign of abnormal (high) LV filling pressure.

Speckle-tracking analysis was conducted off-line by an independent experienced examinator who was blinded to the patients’ clinical data and outcomes (M.B.). All parameters were averaged over 3 cardiac cycles. The LV and LA endocardial border was automatically traced using commercially available software (AFI, AFI LA, GE Medical Systems) and was manually adjusted by the performing physician. End-systole was defined by aortic valve closure. LV GLS value was measured from the average of the A4C, A2C, and apical long axis (3-chamber) views. R-LAS value was measured from the average of A4C and A2C views. Abnormal (reduced) LV GLS was defined as <−18%, and abnormal R-LAS was defined as <−35% [1].

### 2.3. Statistical Analysis

In the first step of statistical analysis, all continuous data were checked for normality using the Shapiro–Wilk test. Based on the results of this analysis, continuous variables were reported as means ± standard deviations (for data with normal distribution) or medians and interquartile ranges (for data with asymmetrical distribution). Categorical variables were reported as number of cases (N) or % of cases. Differences in continuous variables between studied groups were checked using the *t*-test (for normal distribution) or u-test (for asymmetrical one). Differences in categorical variables were assessed with the chi-squared test. Statistical analysis was performed with statistical software Statistica version 5.0 (StatSoft, Tula, OK, USA).

## 3. Results

### 3.1. Patients and Controls

During the study period, 16 patients with acute decompensation of chronic heart failure met the study inclusion criteria (patient group), and 16 individuals were recruited to the control group. The basic demographics of the patients and controls are reported in Table 1. In the patient group, the HFrEF and HFpEF subgroups consisted of six and ten patients, respectively.

Comparing the patients with the controls, the controls were younger and had lower levels of serum creatinine and NT-proBNP but did not differ in body composition (body mass index = BMI).

Looking at the differences between HFrEF and HFpEF patients, no differences in age, estimated renal function, liver enzymes, prevalence of atrial fibrillation and NT-proBNP levels were found. Additionally, there were no significant differences in B-blockers, diuretics and digoxin on admission and upon release. However, on admission, HFrEF patients were more frequently treated with angiotensin-converting enzyme inhibitors, angiotensin 1 receptor blockers or angiotensin receptor/neprilysin inhibitors, mineralocorticoid receptor antagonists and sodium-glucose cotransporter-2 inhibitors (Table 1).

### 3.2. 2D Speckle-Tracking Analysis

**Figure 1:**
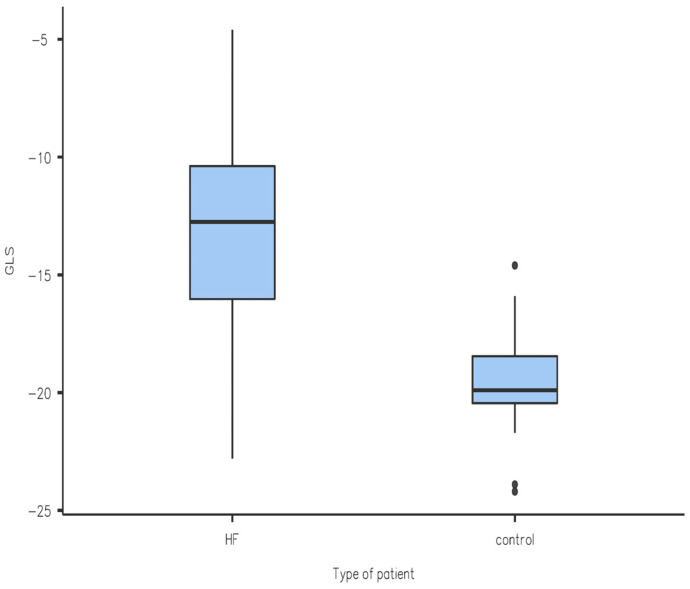
Comparison of LV-GLS between patients with heart failure (HF) and control group.

**Figure 2:**
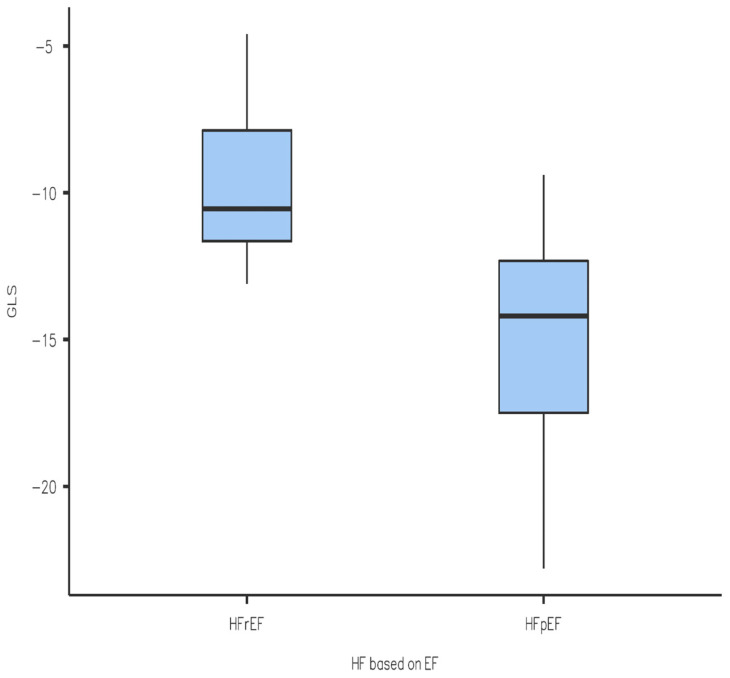
Comparison of LV-GLS between HFrEF and HFpEF subgroups. LV-GLS—left ventricular global longitudinal strain, HF—heart failure; HFrEF—heart failure with reduced left ventricular ejection fraction; HFpEF—heart failure with preserved left ventricular ejection fraction.

**Figure 3:**
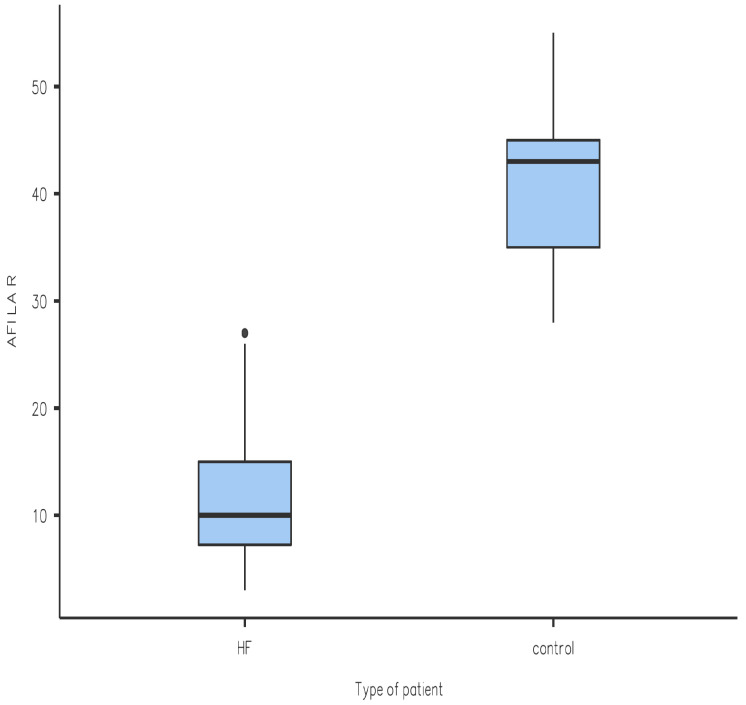
Comparison of R-LAS between patients with HF and control group. HF—heart failure; R-LAS—reservoir longitudinal atrial strain.

**Figure 4:**
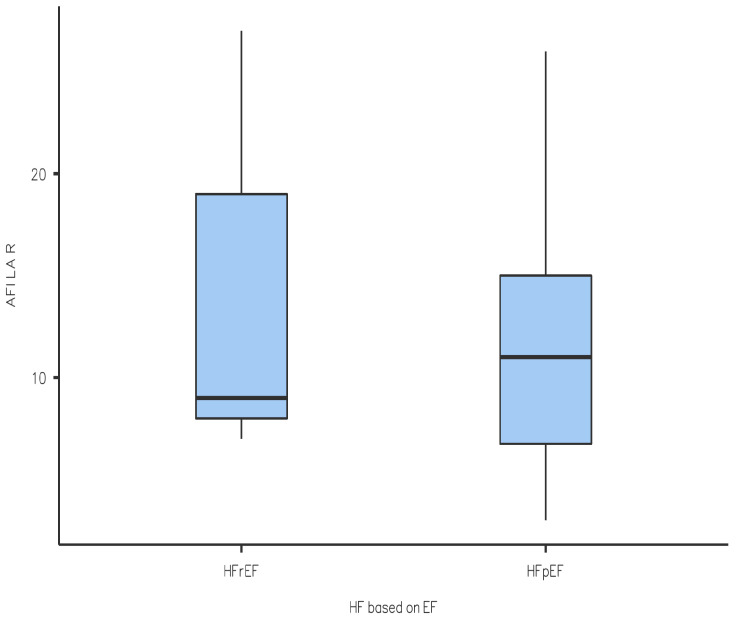
Comparison of R-LAS between HFrEF and HFpEF subgroups. HF—heart failure; HFrEF—heart failure with reduced left ventricular ejection fraction; HFpEF—heart failure with preserved left ventricular ejection fraction; R-LAS—reservoir longitudinal atrial strain.

We performed a Student *t*-test statistical analysis of the results obtained, which yielded the following results (also seen in a graphical form in Figure 1, Figure 2, Figure 3 and Figure 4): the values of LV-GLS and R-LAS were significantly lower in both the HFrEF and HFpEF subgroups in comparison with the control group (LV-GLS: −13.4 ± 4.7% vs. −19.7 ± 2.5%, *p* ˂ 0.05; R-LAS: +12.2 ± 6.9% vs. +40.3 ± 7.4%, *p* ˂ 0.05). Furthermore, there was a significant difference in LV-GLS (−9.6 ± 3.2% vs. −15.2 ± 4.3%, *p* ˂ 0.05) but not in R-LAS (+13.7 ± 8.6% vs. +11.4 ± 6.2%) between the HFrEF and HFpEF subgroups (Figure 1, Figure 2, Figure 3 and Figure 4).

## 4. Discussion

In this study, we aimed to compare LV-GLS and R-LAS results between patients with acute decompensation of HF and individuals with no known cardiovascular disease. Treatment consisted of standard therapy with loop diuretics with titration to effect to alleviate symptoms and signs of congestion, alongside the optimization of evidence-based pharmacological therapy to reduce mortality and rehospitalization for HF where possible as recommended by the 2021 European Society of Cardiology guidelines [6]. We found that LV-GLS was significantly reduced in patients hospitalized for acute decompensation of HF in both the HFrEF and HFpEF subgroups compared to the control group. In particular, the reduction in LV-GLS in the HFpEF group is an interesting finding (Figure 2) and it is in line with a study performed by Bshiebish et al. [9] that found a reduced LV-GLS in patients with HFpEF indicating the presence of left ventricular systolic dysfunction (reduced longitudinal contractility) despite preserved EF. Also of note is the result that R-LAS was significantly reduced in both the HFpEF and HFrEF subgroups compared to the control group, which can be explained by the fact that LA experiences hypertension due to increased filling pressures in LV, and indeed dysfunction, in patients with HF regardless of EF [10]. Interestingly, we observed no significant difference in R-LAS between the HFpEF and HFrEF subgroups, which is an opposite finding to that of a systematic review conducted by Jin et al. [9], which found that R-LAS was markedly reduced in patients with HFrEF in comparison with HFpEF. As mentioned, R-LAS seems to be a potent and sensitive marker of impaired LV filling (increased LV filling pressure) [3]. Our observation of reduced R-LAS in patients with HFrEF indirectly implies that impaired diastolic LV filling also plays an important role in acute decompensation of HF in patients with HFrEF, and that the pathology of decompensated HFrEF is more complex and does not include reduced global (and/or regional) systolic function only. However, looking at the available data, there is only a limited number of studies addressing this issue; therefore, it is difficult to offer any final explanation. In a previous study on patients with acute decompensated HFrEF, Deferm et al. found that R-LAS was significantly reduced at baseline and improved with decongestion [11], which supports our observation. Nevertheless, their study included only patients with HFrEF, and the study population included only 31 patients. In another study, in patients with non-ischemic dilated cardiomyopathy, R-LAS values were significantly lower in those who experienced adverse clinical events (ventricular arrhythmia or acute decompensated HF) [12]. Unfortunately, there is no study addressing the issue of R-LAS in patients with acute decompensated HFpEF which would allow for a comparison of the results. Summarizing, although R-LAS seems to be significantly reduced in patients with acute decompensated HF (irrespective of LV EF), right now the role of the assessment of R-LAS in clinical practice is not fully determined, and the role of R-LAS in the management of acute decompensated HF needs to be clarified in further research.

In addition, right now, it is not entirely clear how our findings implicate clinical practice, as there is no direct therapeutic approach affecting impaired LV filing or dysfunction of longitudinal LV contractility available. However, one can speculate that future research will develop therapeutic approaches for these conditions. For example, Katogiannis et al. showed that decreased LAS was improved in metformin-treated patients with type 2 diabetes after 6-month-long treatment with liraglutide, empagliflozin or their combination (compared to insulin therapy) [13]. One can therefore speculate that the clinical benefit of the early onset of SGLT-2 inhibitors in acute decompensated HF might be seen, at least in part, due to improved left ventricular filling; thus, one might advocate for the early start of modern HF therapy in acute decompensated HF. However, further studies are needed for clarification.

Another issue is the issue of possible limitations of using 2D ST assessment in patients with acute decompensated HF. As mentioned, 2D ST needs sufficient imaging quality, and this can be difficult to achieve in settings of acute decompensated HF (usually due to dyspnea and intolerance of horizontal patient position). Thus, the use of 2D ST analysis in clinical practice might be limited, and in a portion of acute decompensated HF patients, it would be not possible to obtain the data from 2D ST analysis. This would be probably the most important clinical limitation of the method.

## 5. Limitations

Firstly, this study’s sample size is relatively small, which in part is due to the fact that 2D ST requires precise measurements and suitable images from A4C, A3C and A2C projections, which depend on the patient’s habitus and also other factors. Therefore, only patients in whom echocardiography allowed us to obtain images usable for 2D ST analysis were enrolled in our study. This might have caused selection bias. Therefore, our results can probably only be applied to acute HF patients with good echocardiographic imaging quality for 2D ST analysis and probably cannot be generalized to all acute decompensated HF patients. Secondly, the measurements were not all performed by one cardiologist (but by two individuals), so there is inevitable room for inter-individual variability in recording and interpreting the echocardiographic data. However, 2D ST analysis was performed by one individual to reduce the risk of inter-individual variability in this analysis. Thirdly, there was an age difference between the control group and the HF groups, and there is a possibility that control individuals will develop HF in future. Fourthly, we did not perform control echocardiography after decongestion. Finally, discretion regarding therapy for acute decompensated heart failure was left to the attending physician. In fact, there were differences in heart failure pharmacotherapy on admission and upon release in patients with HFrEF and HFpEF. These differences in guideline-recommended therapy match current treatment differences for HFrEF and HFpEF and were also caused by the drug administration policy which was valid in our country at the time of the study (for example, at that time, SGLT-2 inhibitors could not be administered in patients with HFpEF). All these facts should be taken into consideration when the results of our study are interpreted.

## 6. Conclusions

Our study demonstrated a significant difference in LV-GLS and R-LAS in all enrolled HF patients compared to the control group. There was also a significant difference in LV-GLS between the HFrEF and HFpEF subgroups. Further studies and larger sample sizes will be required to support these results.

## Figures and Tables

**Table 1:** Basic review of selected laboratory parameters, comorbidities and heart failure therapies in HFrEF and HFpEF subgroups of patients with acute decompensation of chronic heart failure and in control group.

	HFrEF Subgroup	HFpEF Subgroup	Control Group
Number of patients (men/women)	6 (5/1)	10 (6/4)	16 (13/3)
Age	71 (56–78)	76 (64–84)	32 (20–54)
Beta-blockers at admission/upon release (%)	83.3/100	80/80	0
ACE inhibitors, AT1RB, ARNI at admission/upon release (%)	33.3/66.7	70/80	0
MRA at admission/upon release (%)	83.3/100	30/70	0
SGLT2i at admission/upon release (%)	16.7/16.7	0/0	0
CRT at admission/upon release (%)	0/0	0/0	0
Digoxin at admission/upon release (%)	33.3/33.3	30/30	0
Loop diuretics at admission/upon release (%)	83.3/100	60/100	0
BMI (kg/m^2^)	28 ± 5.4	26.7 ± 4.3	24.9 ± 3.5
Serum creatinine (µmol/L)	100.7 ± 35.3	133.7 ± 117.5	80.3 ± 11.2
GFR calculated using Cockroft–Gault equation (mL/min/1.73 m^2^)	67.5 ± 23.2	58.3 ± 31.7	N/E
ALT (µkat/L)	0.47 ± 0.28	0.38 ± 0.24	N/E
AST (µkat/L)	0.61 ± 0.30	0.53 ± 0.25	N/E
NT-proBNP (pg/mL)	7153.1 ± 7254.4	8453.2 ± 6032.0	46.0 ± 9.4
Hemoglobin (g/L)	130.6 ± 15.9	115.3 ± 8.9	N/E
Total serum protein (g/L)	60.2 ± 15.8	63.1 ± 8.1	N/E
EF LV (%)	30.5 ± 9.2	50.3 ± 6.9	58.6 ± 1.1
Myocardial revascularization (%)	50	33	0
History of MI (%)	66.7	33	0
Atrial fibrillation (%)	50	60	0
Valve disease—moderate to severe (%)	83.3	50	0

HFrEF—heart failure with reduced ejection fraction, HFpEF—heart failure with preserved ejection fraction, ACE—angiotensin converting enzyme, AT1RB—angiotensin 1 receptor blocker, ARNI—angiotensin receptor/neprilysin inhibitor, MRA—mineralocorticoid receptor antagonist, N/E—not estimated, SGLT2i—sodium-glucose cotransporter-2 inhibitor, CRT—cardiac resynchronization therapy, BMI—body mass index, GFR—glomerular filtration rate, ALT—alanine aminotransferase, AST—aspartate aminotransferase, NT-proBNP—N-terminal prohormone of brain natriuretic peptide, EF LV—ejection fraction of left ventricle, MI—myocardial infarction.

## Data Availability

All data are available from the corresponding author upon reasonable request.
